# The impact of adverse childhood experiences on sensory thresholds in adults living with multimorbidity and chronic pain: an observational feasibility study

**DOI:** 10.1016/j.bjao.2026.100545

**Published:** 2026-03-10

**Authors:** Dhaneesha N.S. Senaratne, Blair H. Smith, Tim G. Hales, Louise Marryat, Lesley A. Colvin

**Affiliations:** 1 Chronic Pain Research Group, Division of Population Health & Genomics, School of Medicine, University of Dundee, Dundee, UK; 2 Institute of Academic Anaesthesia, Division of Neuroscience, School of Medicine, University of Dundee, Dundee, UK; 3 School of Health Sciences, University of Dundee, Dundee, UK

**Keywords:** adverse childhood experiences, chronic pain, conditioned pain modulation, multimorbidity, multiple long-term conditions, quantitative sensory testing

## Abstract

**Background:**

Epidemiological studies have linked adverse childhood experiences (ACEs) to multimorbidity and chronic pain. One possible mechanism may be altered sensory processing, which could influence symptom development and persistence, and can be assessed by psychophysical methods such as quantitative sensory testing (QST). In this single-site feasibility study, we evaluated a study protocol to examine these relationships. Our primary aim was to assess the feasibility and acceptability of the study procedures. Our secondary aim was to generate preliminary data exploring relationships between ACE exposure and QST parameters.

**Methods:**

This was a single-site feasibility study with a cross-sectional design. Adult participants completed questionnaires (including a 20-item ACE questionnaire), static QST based on the German Research Network on Neuropathic Pain protocol, and dynamic QST (conditioned pain modulation) using pressure and heat test stimuli.

**Results:**

Of 101 people directly approached, 60 were recruited (recruitment rate, 59.4%; 73.3% female [*n*=44]; mean [range] age, 48.8 [19–87] yr). Study completion rate was 100%, and all participants reported that the protocol was either ‘completely acceptable’ (93.3%, *n*=56) or ‘acceptable’ (6.7%, *n*=4). In exploratory analyses, higher ACE exposure was associated with higher odds of chronic pain, multimorbidity, greater medication use, and higher pain severity and interference scores. Higher ACE count was also linked to some mechanical static QST parameters, but there was no relationship with dynamic QST parameters.

**Conclusions:**

This study demonstrated that assessing sensory processing in relation to ACEs among adults with chronic pain and multimorbidity was both feasible and acceptable. Feasibility metrics and preliminary effect estimates will inform protocol refinement, outcome prioritisation, sample size calculations, and recruitment timelines for a future appropriately powered definitive study.

Adverse childhood experiences (ACEs) are increasingly recognised as important factors influencing long-term health. They are defined as potentially stressful events or environments that an individual may be exposed to before the age of 18 yr, and encompass various forms of abuse (e.g. physical), neglect (e.g. emotional), household challenges (e.g. parental separation), and external challenges (e.g. bullying). ACEs are remarkably common, with 61% of adults worldwide reporting at least one, and 16% reporting four or more.^1^ Epidemiological studies link ACEs to poor outcomes in a range of physical health, mental health, and social domains in later life, often in a dose-dependent manner.^2^, ^3^, ^4^, ^5^, ^6^, ^7^, ^8^, ^9^, ^10^, ^11^, ^12^

The mechanisms by which ACEs may influence long-term outcomes are complex, but may involve factors at the epigenetic level (such as altered epigenetic age),^13^^,^^14^ at the cellular level (such as mitochondrial dysfunction),^15^^,^^16^ at the physiological level (such as the inflammatory and stress responses),^17^, ^18^, ^19^ or at the behavioural level (such as sleep disruption).^20^ There is also evidence to suggest that ACEs may disrupt endogenous analgesic systems, which may lead to altered analgesic responses.^19^^,^^21^^,^^22^

Among the health outcomes associated with ACEs, chronic pain and multimorbidity are particularly important. Chronic pain, defined as pain that persists or recurs for longer than 3 months,^23^ has an estimated worldwide point prevalence of 10–50%,^24^, ^25^, ^26^ with 5–12% reported to have ‘high-impact’ pain that interferes significantly with their daily life.^27^^,^^28^ Multimorbidity, defined as the co-occurrence of two or more long-term conditions (LTCs) in the same individual, has a lifetime prevalence of 33% and this is expected to increase as global populations age.^29^ Chronic pain and multimorbidity are closely interrelated; studies from the UK Biobank reported that pain was a common component of disease burden in people living with multimorbidity,^30^ and the likelihood of experiencing pain was greater in people reporting more LTCs.^31^ Interestingly, the type of chronic pain also appeared to vary with the number of LTCs: compared with those with no LTCs, people with more than four LTCs were over three times more likely to have any form of chronic pain, but more than 20 times more likely to have widespread chronic pain.^31^ These findings raise the possibility that multimorbidity itself may reflect underling alterations in sensory processing, making it a valuable context for exploring sensory mechanisms linked to ACEs.

Quantitative sensory testing (QST) offers a means of investigating sensory processing through structured assessment of the somatosensory system. It is a psychophysical method that incorporates both the physical properties and the psychological perception of a stimulus. QST may be static or dynamic; static QST assesses baseline sensory function whereas dynamic QST assesses mechanisms of sensory processing by using more complex stimulation protocols.^32^^,^^33^ QST is frequently used in chronic pain research, but to our knowledge, it has not been applied in the context of multimorbidity. Given the potential for shared neurobiological pathways linking ACEs, pain, and chronic illness (such as inflammation, neuroendocrine dysregulation, and central sensitisation), it is plausible that different QST patterns may be present in people with different numbers of LTCs.

The literature examining the association between ACEs and QST findings is inconsistent,^34^, ^35^, ^36^, ^37^, ^38^, ^39^, ^40^ possibly owing to the heterogeneity of ACE assessments and QST methodology. On the basis of previous evidence linking ACEs with chronic pain^3^^,^^8^ and multimorbidity,^11^ we hypothesised that individuals with greater ACE exposure would exhibit ‘gain of function’ in QST parameters (i.e. higher sensitivity to physical stimuli).

Thisstudy was an observational feasibility study conducted to inform the design of a future, larger investigation into the impact of ACEs on sensory processing in people living with Multimorbidity And/or chronic Pain (ACE-MAP study). Our primary aim was to assess the feasibility and acceptability of the proposed study procedures. Our secondary aim was to generate preliminary data exploring potential relationships between ACE exposure and QST parameters. This would help inform protocol refinement, outcome prioritisation, sample size calculations, and recruitment timelines for a future robust appropriately powered definitive study.

## Methods

The protocol for this study has been published previously, with a detailed description of the study design and procedures.^41^ A summary is provided below.

### Consortium Against Pain inEquality

This study is part of a broader multi-institution collaboration, the Consortium Against Pain inEquality (CAPE).^42^ CAPE consists of a number of scientists, clinical researchers, pain specialists, epidemiologists, psychologists, and people with lived experience of chronic pain who together are investigating the relationship between ACEs and chronic pain in adulthood.

### Ethical approval

This study was approved by the National Health Service Scotland B Research Ethics Committee (reference: 24/SS/0031) and pre-registered in the ISRCTN registry (ID: ISRCTN10049430). It was conducted in concordance with the principles of the Declaration of Helsinki (2013) and Good Clinical Practice. All participants provided written consent to take part, and were informed that they could withdraw from the study at any point.

### Study design and recruitment

This was a single-site feasibility study with a cross-sectional design. Potential participants were identified through multiple streams: the Scottish Health Research Register and Biobank (SHARE) database,^43^ the NHS Tayside Chronic Pain Service, advertising materials (study posters, website content), and word of mouth. Potential participants were provided with either a paper or electronic participant information sheet (depending on individual preference) and were given a minimum of 24 h to consider their participation.

Inclusion criteria were:

• Age ≥ 18 yr.

Exclusion criteria were:

• Lacking capacity to consent to participate in the study.
• Unable to read or speak English to a sufficient standard to complete questionnaires or to follow instructions for psychophysical testing.

We aimed to recruit participants to four study groups: (1) chronic pain with multimorbidity, (2) chronic pain without multimorbidity, (3) multimorbidity without chronic pain, and (4) a control group (i.e. people with neither multimorbidity nor chronic pain). Multimorbidity was defined as the presence of two or more LTCs, using a list of LTCs identified by Ho and colleagues^44^ in a Delphi consensus study on multimorbidity measurement.

When considering sample size, Whitehead and colleagues^45^ suggested that 10 per treatment arm in the pilot phase would provide enough preliminary data to determine the sample size needed to detect a medium effect in a full interventional trial. Although this is not an interventional study, we believed the principles to be transferable and set a target to recruit 40 participants in total, with 10 in each group. This target was above the median sample size for feasibility studies registered in the ISRCTN registry.^46^ However, during recruitment, it became apparent that the four groups were unequally represented, despite pre-screening and targeted over-recruitment to 60 participants. Recruitment was planned to run from July 2024 to December 2025 (based estimated recruitment rate and investigator availability), but terminated in February 2025 after this 50% over-recruitment target was reached.

### Study procedure

Participants attended one assessment session (lasting 1.5–2 h) at Ninewells Hospital and Medical School, University of Dundee, UK. At this session, participants completed the consent process (including signing a written consent form), completed a range of questionnaires, and then engaged in QST.

The questionnaires covered patient characteristics, health behaviours, LTCs (using the list of LTCs identified by Ho and colleagues^44^), chronic pain (using the UK Biobank Pain Web Questionnaire^47^), ACEs (using the new CAPE ACE questionnaire), and concurrent medication.

The CAPE ACE questionnaire was co-produced with the CAPE patient and public involvement (PPI) group after a systematic review of existing validated questionnaires.^48^ Three existing validated questionnaires (the Adverse Childhood Experiences International Questionnaire [ACE-IQ],^49^ the Trauma and Distress Scale [TDS],^50^ and the Traumatic Experiences Checklist [TEC]^51^) were selected and adapted following PPI feedback. This included ensuring that the wording was respectful and acceptable to people who have experienced ACEs (and to those who have not), and that a broad and relevant range of ACEs were included to reflect a range of backgrounds and potentially stressful experiences. It also involved adaptation to ensure that the questionnaire was suitable for completion directly by a participant, in the absence of an interviewer or administrator. The questionnaire also provided information and contact details for additional support, in case the issues raised caused distress. The CAPE ACE questionnaire measures 20 ACEs across four domains. It is undergoing formal validation in a parallel process and the final questionnaire will be available for use in a future definitive study.

We performed static QST using a protocol devised by the German Research Network on Neuropathic Pain (DFNS), which measures cold detection threshold (CDT), warm detection threshold (WDT), thermal sensory limens (TSL), paradoxical heat sensations (PHS), cold pain threshold (CPT), heat pain threshold (HPT), mechanical detection threshold (MDT), mechanical pain threshold (MPT), mechanical pain sensitivity (MPS), dynamic mechanical allodynia (DMA), wind-up ratio (WUR), vibration detection threshold (VDT), and pressure pain threshold (PPT).^52^^,^^53^ These measurements were taken bilaterally on the hand or forearm and values were combined, as per the DFNS protocol. In keeping with standard DFNS QST nomenclature, increased sensitivity to stimuli was described as ‘gain of function’ and decreased sensitivity as ‘loss of function’. We performed dynamic QST with the cold water conditioned pain modulation (CPM) paradigm: the participant submerged their non-dominant hand in a cold water bath at 10^o^C for as long as they could tolerate, up to a total of 2 min (the conditioning stimulus), and had HPT followed by PPT (the test stimuli) measured on the dominant hand. All sensory testing was performed in the same testing environment, by one investigator (DNSS), taking care to ensure consistency in approach, language, and measurement.

Finally, participants completed questions on study acceptability, using a questionnaire adapted from Sekhon and colleagues.^54^ Participants were offered a £25 gift voucher and reimbursement of their travel expenses for taking part.

A detailed description of the study procedure, including the equipment used and descriptions of how individual QST tests were performed, can be found in the published study protocol.^41^

### Statistical analysis

Patient characteristics and questionnaire data were reported using counts (percentage) for categorical data and median (range) for continuous data. The primary aim was to assess the feasibility and acceptability of the proposed study procedures. Recruitment feasibility was assessed by calculating the overall recruitment rate (number of participants successfully recruited per week) and the recruitment proportion (number of participants successfully recruited divided by the number of potential participants approached). The target minimum recruitment rate was one participant every 2 weeks (0.5 participants per week). Acceptability parameters were reported using counts (percentage).

The secondary aim was to generate exploratory data to understand the impact of ACEs on QST thresholds in people with chronic pain, multimorbidity, or both. The number of ACEs reported through answers to the CAPE ACE questionnaire were summed to generate an ACE count (potential range 0–20). Static QST data collected using the DFNS protocol were analysed as both raw values and as age- and gender-matched *z*-scores using DFNS reference data from healthy controls.^52^^,^^53^^,^^55^ *z*-Scores were calculated using the following formula^52^^,^^55^:

$$z-score=\frac{\left({value}_{participant}-{mean}_{reference}\right)}{{SD}_{reference}}$$

*z*-Scores for HPT, MPT, and PPT were multiplied by −1 so that all positive *z*-scores reflected gain of function (higher sensitivity to stimuli) and all negative scores reflected loss of function (lower sensitivity to stimuli). No participants reported PHS, so this parameter was removed from the data presented here. Dynamic QST data (CPM) for HPT and PPT were reported as both absolute CPM and percentage CPM using the following formulae^56^:

$$absolute\;CPM={value}_{before\;conditioning\;stimulus}-{value}_{after\;conditioning\;stimulus}$$

$$percentage\;CPM=\frac{{value}_{before\;conditioning\;stimulus}-{value}_{after\;conditioning\;stimulus}}{{value}_{before\;conditioning\;stimulus}}$$

Thus, a positive CPM value indicates higher pain sensitivity (facilitatory response), whereas a negative CPM value indicates lower pain sensitivity (inhibitory response).^56^

Unadjusted exploratory analyses between number of ACEs and QST parameters were performed using scatter plots, reporting Pearson’s correlation coefficient (*r*). Adjusted exploratory analyses were guided by casual inference principles. Our analytical framework is represented in a directed acyclic graph (DAG) (Supplementary Fig. 1), a diagrammatic representation of the assumed causal structure, which informed the selection of the variables for adjustment.^57^^,^^58^ For the primary analyses with ACEs as the exposure, the adjustment set was age, sex at birth, ethnicity, and childhood socioeconomic status (SES). For sensitivity analyses with chronic pain status as the exposure, ACEs were additionally included in the adjustment set. For sensitivity analyses with multimorbidity as the exposure, both ACEs and chronic pain status were added to the adjustment set. Education level was used as a proxy for childhood SES.^59^ Bias adjusted logistic regression models using Firth’s penalised logistic regression were used for binary outcomes (as standard maximum-likelihood logistic regression models can become unstable with small sample sizes),^60^ and are reported using the odds ratio (OR) and 95% confidence intervals (CIs). Linear regression models were used for continuous outcomes, and are reported using the beta coefficient (*β*), standard error, and 95% CIs for the beta coefficient. The *β* coefficient reflects the degree of change in the outcome variable given a unit change in the exposure variable (i.e. the change in the outcome variable given a +1 increase in ACE count). Both unadjusted and adjusted analyses were considered hypothesis-generating, and so *P*-values are not presented.

All statistical analyses were performed in R v4.5.1 using the RStudio integrated development environment (RStudio Team, Boston, MA, USA).

### Patient and public involvement

This study has had input from members of our PPI group, throughout the research cycle.^42^ This group consists of eight individuals with lived experiences of ACEs, multimorbidity, and chronic pain. The group provided valuable input into the development of the research question and helped shape key elements of the study design, including consideration of participant burden and the acceptability of the proposed procedures. Group members reviewed all participant-facing materials, offering specific feedback on clarity, tone, and accessibility. They highlighted the importance of ensuring that participants understood why the study was being conducted, and emphasised the need for clear, transparent communication. The group strongly supported the use of financial reimbursement as recognition of participants’ time and effort, recommending a figure in the range of £15–25. They also advised that participants may find it difficult to discuss sensitive personal experiences in person, particularly with unfamiliar researchers, and recommended offering a choice of data collection formats (e.g. paper-, tablet-, or laptop-based questionnaires) to accommodate different physical and emotional needs. This input was instrumental in refining the study protocol and ensuring it was acceptable and accessible to the intended population.

## Results

### Feasibility and acceptability

**Fig 1:**
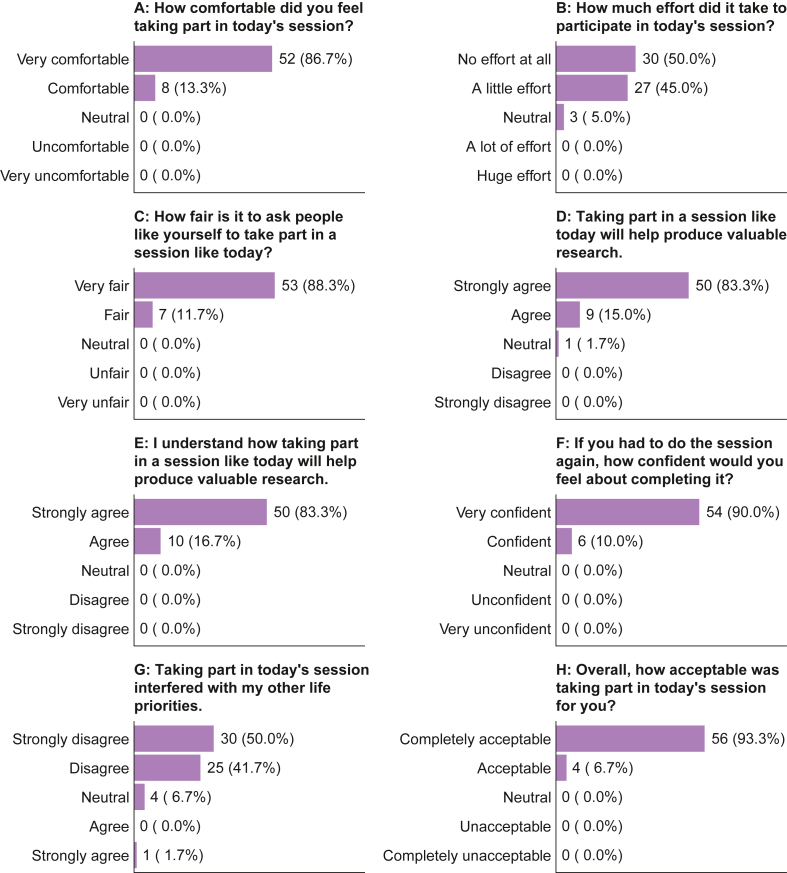
Participant responses to the study acceptability questionnaire.

Between July 2024 and February 2025, 101 potential participants were directly approached and 60 were enrolled (59.4%). Recruitment averaged two participants per week, exceeding the minimum target of 0.5 per week, and surpassed the participant target of 40, all within the first half of the planned recruitment window (July 2024 to December 2025). All participants completed all aspects of the study protocol, with no missing data in any study domain, and no participant withdrawals. Acceptability was high: 93.3% (*n*=56) rated the protocol ‘completely acceptable’ and 6.7% rated it ‘acceptable’ (*n*=4) (Fig. 1). However, despite targeted over-recruitment, we were not able to recruit equally to the originally proposed study groups (chronic pain with multimorbidity=36, chronic pain without multimorbidity=2, multimorbidity without chronic pain=7, and control=15). We therefore did not perform our planned per-group analyses.

### Participant characteristics

Sixty participants were recruited. The median age was 50 yr (range, 19–87 yr) and 73.3% (*n*=44) were female. Participant characteristics are shown in Table 1.

**Table 1:** Participant characteristics. Classification of analgesic medications was based on chapters of the British National Formulary.^61^ LTC, long-term condition; SIMD2020, Scottish Index of Multiple Deprivation 2020.

	Study sample
	(*n*=60)
Age, mean (range), yr	48.8 (19–87)
Sex assigned at birth, *n* (%)	
Female	44 (73.3)
Male	16 (26.7)
Ethnicity, *n* (%)	
White	54 (90.0)
Asian or Asian British	4 (6.7)
Arab or Arab British	1 (1.7)
Mixed or multiple ethnic groups	1 (1.7)
Highest level of education, *n* (%)	
Secondary school	11 (18.3)
Vocational qualification or equivalent	13 (21.7)
University qualification or equivalent	35 (58.3)
Prefer not to answer	1 (1.7)
Household income, *n* (%)	
£0–£17 999	6 (10.0)
£18 000–£30 999	13 (21.7)
£31 000–£51 999	11 (18.3)
£52 000–£100 000	19 (31.7)
Over £100 000	7 (11.7)
Prefer not to answer	4 (6.7)
SIMD2020 quintile, *n* (%)	
1: Most deprived	11 (18.3)
2	4 (6.7)
3	14 (23.3)
4	21 (35.0)
5: Least deprived	10 (16.7)
Number of LTCs, median (range)	2 (0–12)
Number of regular medications, median (range)	3 (0–13)
Regular analgesic medications	
Total, median (range)	0 (0–5)
Gabapentinoids, *n* (%)	3 (5.0)
Non-gabapentinoid anti-epileptics, *n* (%)	3 (5.0)
Non-opioid analgesics, *n* (%)	10 (16.7)
Non-steroidal anti-inflammatory drugs, *n* (%)	1 (1.7)
Opioid analgesics, *n* (%)	5 (8.3)
Serotonin–norepinephrine reuptake inhibitors, *n* (%)	8 (13.3)
Tricyclic antidepressants, *n* (%)	9 (15.0)

### Adverse childhood experiences

Most participants (98.3%, *n*=59) reported having experienced at least one ACE, with a median number of 6 (range, 0–19). The most commonly reported ACEs were bullying (71.7%, *n*=43), emotional abuse (70.0%, *n*=42), and community violence (61.7%, *n*=37). The prevalence of individual ACEs is shown in Supplementary Figure 2.

### Chronic pain

Chronic pain was reported by 63.3% of participants (*n*=38). Of these, 15.8% (*n*=6) reported a duration of 3–12 months, 18.4% (*n*=7) reported 1–5 yr, and 65.8% (*n*=25) reported more than 5 yr. The most commonly reported pain sites were back (36.8%, *n*=14), neck (31.6%, *n*=12), and ‘all over’ (31.6%, *n*=12). In exploratory regression analyses, higher ACE count was linked with higher odds of chronic pain (OR, 1.24; 95% CI, 1.03–1.55) and a greater number of regular analgesic medications (*β*=0.10, se=0.04; 95% CI, 0.03–0.17). Higher ACE count was also related to higher participant-reported pain severity and pain interference scores (Table 2).

**Table 2:** Impact of ACE count on participant-reported pain severity and pain interference scores. Each line reports a different linear regression model adjusted for age, sex at birth, ethnicity, and education (covariate model parameters not shown). ACE, adverse childhood experience; *β*, beta regression coefficient; CI, confidence interval.

	*β*	se	95% CI
Pain severity			
Lowest in last 7 days	0.26	0.07	0.13–0.39
Average in last 7 days	0.28	0.06	0.16–0.41
Highest in last 7 days	0.27	0.08	0.11–0.42
Now	0.37	0.05	0.28–0.47
Pain interference with…			
General activity	0.40	0.13	0.15–0.65
Mood	0.46	0.09	0.27–0.64
Ability to walk	0.48	0.12	0.24–0.73
Work	0.45	0.11	0.23–0.67
Relationships	0.38	0.11	0.16–0.59
Sleep	0.47	0.12	0.23–0.71
Enjoyment of life	0.39	0.11	0.17–0.61

### Multimorbidity

Multimorbidity was reported by 60.0% of participants (*n*=36). The median number of LTCs was 2 (range, 0–12). The most commonly reported LTCs were depression (33.3%, *n*=20), anxiety (30.0%, *n*=18), and asthma (20.0%, *n*=12). In exploratory regression analyses, higher ACE count was linked with higher odds of multimorbidity (OR, 1.35; 95% CI, 1.12–1.72), a greater number of LTCs (*β*=0.36, se=0.07; 95% CI, 0.22–0.50), and a greater number of regular medications (*β*=0.37, se=0.10; 95% CI, 0.17–0.57).

### Quantitative sensory testing

**Fig 2:**
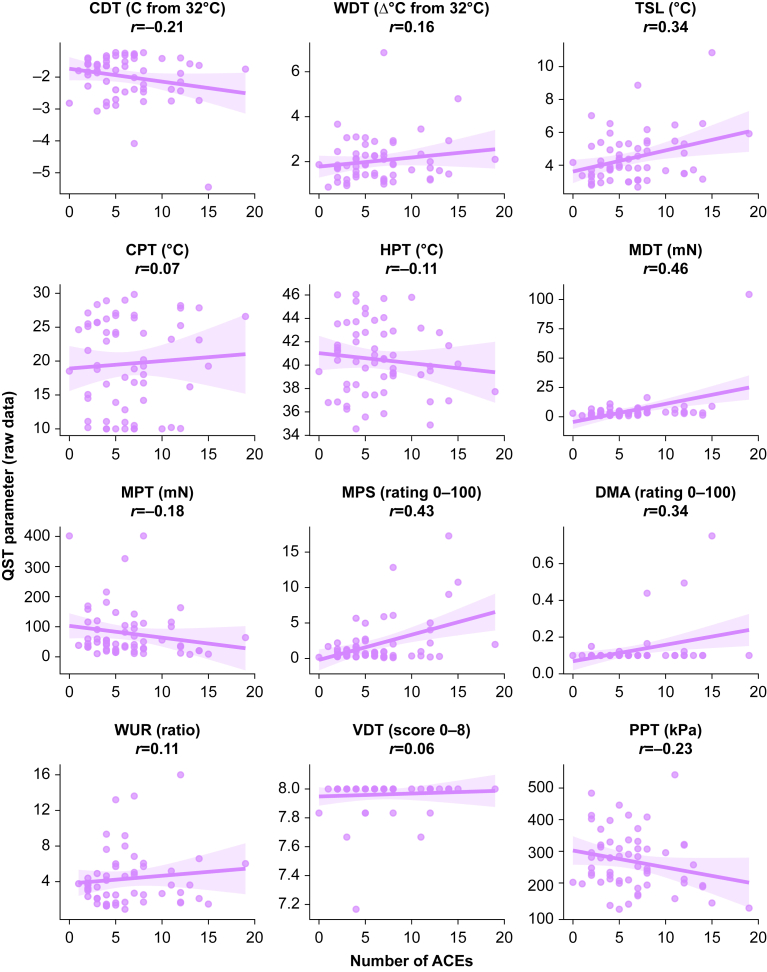
Hypothesis-generating exploratory data showing the impact of number of ACEs reported on static QST parameters, reported as raw values. The shaded area represents the 95% confidence interval for the line of best fit. The Pearson correlation coefficient (*r*) is displayed for each relationship. ACE, adverse childhood experience; CDT, cold detection threshold; CPT, cold pain threshold; DMA, dynamic mechanical allodynia; HPT, heat pain threshold; MDT, mechanical detection threshold; MPS, mechanical pain sensitivity; MPT, mechanical pain threshold; PPT, pressure pain threshold; QST, quantitative sensory testing; TSL, thermal sensory limens; WDT, warm detection threshold; WUR, wind-up ratio; VDT, vibration detection threshold.

Static and dynamic QST protocols were well tolerated, with complete data obtained from all participants. Unadjusted exploratory analyses investigating the relationships between number of ACEs reported and static QST measures are shown in Figures 2 (raw values) and 3 (*z*-transformed values). Moderate correlation was seen for MDT in both raw and *z*-score analyses, and for MPS in raw analyses.

**Fig 3:**
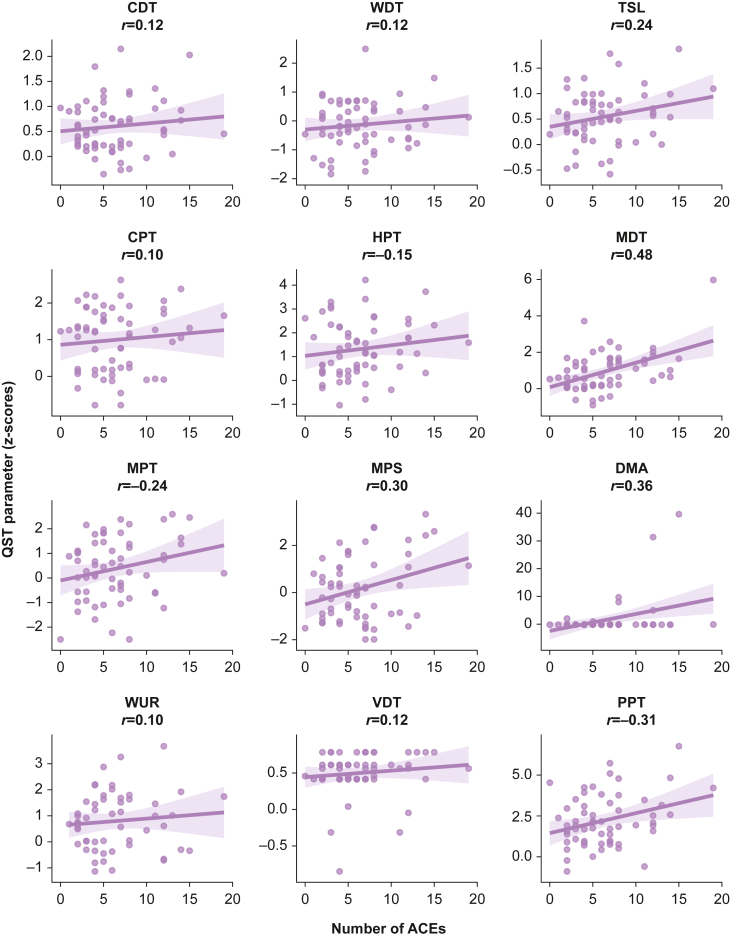
Hypothesis-generating exploratory data showing the impact of number of ACEs reported on static QST parameters, reported as *z*-scores relative to DFNS reference data. Data have been transformed so that a positive *z*-score indicates gain of function (higher sensitivity to stimuli). The shaded area represents the 95% confidence interval for the line of best fit. The Pearson correlation coefficient (*r*) is displayed for each relationship. ACE, adverse childhood experience; CDT, cold detection threshold; CPT, cold pain threshold; DFNS, German Research Network on Neuropathic Pain; DMA, dynamic mechanical allodynia; HPT, heat pain threshold; MDT, mechanical detection threshold; MPS, mechanical pain sensitivity; MPT, mechanical pain threshold; PPT, pressure pain threshold; QST, quantitative sensory testing; TSL, thermal sensory limens; WDT, warm detection threshold; WUR, wind-up ratio; VDT, vibration detection threshold.

In exploratory regression analyses, higher ACE counts appeared to be related to gain of function in MDT, MPS, and DMA in both raw values and *z*-scores (Table 3). Higher ACE counts also showed possible relationships with gain of function in raw TSL values, MPT *z*-scores, and PPT *z*-scores. There did not appear to be any relationship between multimorbidity status or chronic pain status with any static QST parameter (Supplementary Tables 1 and 2, Supplementary material).

**Table 3:** Impact of ACE count on static QST parameters, reported as raw values and *z*-scores. Each line reports two separate linear regression models (for raw values and *z*-scores, respectively) adjusted for age, sex at birth, ethnicity, and education (covariate model parameters not shown). ACE, adverse childhood experience; CDT, cold detection threshold; CI, confidence interval; CPT, cold pain threshold; DMA, dynamic mechanical allodynia; HPT, heat pain threshold; MDT, mechanical detection threshold; MPS, mechanical pain sensitivity; MPT, mechanical pain threshold; PPT, pressure pain threshold; QST, quantitative sensory testing; TSL, thermal sensory limens; WDT, warm detection threshold; WUR, wind-up ratio; VDT, vibration detection threshold. ∗*z*-Scores for HPT, MPT, and PPT were multiplied by −1 so that all positive *z*-scores reflect gain of function (higher sensitivity to stimuli) and all negative scores reflect loss of function (lower sensitivity to stimuli).

QST parameter	Raw values			*z*-Scores		
	*β*	se	95% CI	*β*	se	95% CI
CDT (Δ°C from 32°C)	−0.03	0.02	−0.08 to 0.01	0.01	0.02	−0.02 to 0.05
WDT (Δ°C from 32°C)	0.03	0.03	−0.03 to 0.10	0.02	0.03	−0.03 to 0.08
TSL (°C)	0.10	0.04	0.02–0.19	0.03	0.02	−0.00 to 0.06
CPT (°C)	0.14	0.24	−0.33 to 0.61	0.02	0.03	−0.03 to 0.08
HPT (°C)	−0.10	0.10	−0.30 to 0.09	0.05∗	0.04	−0.02 to 0.12
MDT (mN)	1.42	0.43	0.58–2.27	0.12	0.03	0.05–0.18
MPT (mN)	−5.27	2.71	−10.58 to 0.03	0.10∗	0.04	0.02–0.17
MPS (rating 0–100)	0.42	0.10	0.22–0.63	0.12	0.04	0.04–0.21
DMA (rating 0–100)	0.01	0.00	0.00–0.02	0.65	0.21	0.23–1.07
WUR (ratio)	0.03	0.11	−0.19 to 0.26	0.01	0.04	−0.07 to 0.09
VDT (score 0–8)	0.00	0.00	−0.00 to 0.01	0.01	0.01	−0.01 to 0.03
PPT (kPa)	−4.60	3.13	−10.74 to 1.55	0.10∗	0.05	−0.00 to 0.20

**Fig 4:**
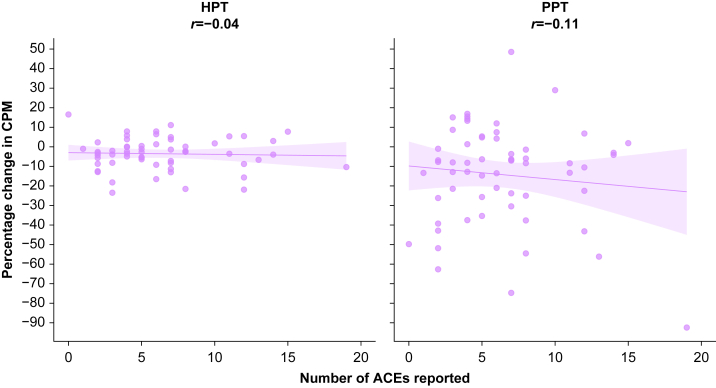
Hypothesis-generating exploratory data showing the impact of number of ACEs reported on dynamic QST parameters (CPM), reported as percentage change relative to baseline. A negative value indicates lower pain sensitivity (inhibitory response), whereas a positive value indicates higher pain sensitivity (facilitatory response). The Pearson correlation coefficient (*r*) is displayed for each relationship. ACE, adverse childhood experience; CPM, conditioned pain modulation; HPT, heat pain threshold; PPT, pressure pain threshold; QST, quantitative sensory testing.

Figure 4 displays the relationship between the number of ACEs reported and dynamic QST (CPM) measures. In exploratory regression analyses, there did not appear to be any relationship between ACE count and absolute CPM or percentage change CPM (Table 4). There was also no apparent relationship between multimorbidity status or chronic pain status and either CPM parameter (Supplementary Tables 3 and 4, Supplementary material).

**Table 4:** Impact of ACE count on dynamic QST, reported as absolute CPM effect and percentage CPM effect. Each line reports two separate linear regression models (for absolute and percentage CPM effects, respectively) adjusted for age, sex at birth, ethnicity, and education (covariate model parameters not shown). ACE, adverse childhood experience; *β*, beta regression coefficient; CI, confidence interval; CPM, conditioned pain modulation; HPT, heat pain threshold; PPT, pressure pain threshold; QST, quantitative sensory testing.

CPM parameter	Absolute CPM effect			Percentage CPM effect		
	*β*	se	95% CI	*β*	se	95% CI
HPT	−0.10	0.11	−0.31 to 0.11	−0.26	0.28	−0.80 to 0.28
PPT	−0.13	2.14	−4.32 to 4.06	−0.73	0.92	−2.53 to 1.06

## Discussion

This feasibility study evaluated a study protocol designed to assess the relationship between ACEs and sensory processing, measured through QST, in adults with chronic pain, multimorbidity, or both. The study procedures were well tolerated, with recruitment and completion rates higher than expected, and positive participant feedback. However, despite a good recruitment rate and targeted recruitment strategies, we were unable to recruit equally to the four study groups in our protocol. Many of the participants identified on screening as belonging to the ‘chronic pain without multimorbidity’ group were subsequently found to have multimorbidity once their study data were analysed. This is perhaps not surprising; given the strong association between chronic pain and multimorbidity^31^; it may be that the population prevalence of people in this group is low. Furthermore, recruitment from a tertiary chronic pain service is likely to identify more complex patients, and so other recruitment strategies (such as through primary care services, or through more targeted pre-screening processes) might be more appropriate to identify individuals in the ‘chronic pain without multimorbidity’ group. Nevertheless, in general, these findings support the feasibility and acceptability of the protocol, and suggest that a larger-scale study is warranted to examine these associations more robustly.

Exploratory analyses involving participant-reported outcomes indicated that higher ACE count was linked to greater odds of multimorbidity, a greater number of LTCs, and a greater number of regular medications, which is in concordance with the conclusion of a recent systematic review and meta-analysis.^11^ Similarly, higher ACE count was also linked to greater odds of chronic pain, a greater number of regular analgesic medications, higher participant-reported pain severity scores, and higher participant-reported pain interference scores, which is in concordance with the conclusions of several recent systematic reviews.^3^^,^^8^^,^^10^ In the wider literature, a higher ACE burden is consistently associated with poor long-term health outcomes^2^^,^^4^^,^^6^ and our results, although exploratory in nature, align with this established relationship.

Exploratory analyses involving static QST parameters were less clear cut. Higher ACE count appeared to be related to gain of function (higher sensitivity) in some mechanical parameters in both unadjusted (MDT and MPS) and adjusted (MDT, MPS, DMA) analyses. The absolute effect sizes (as measured by the β coefficients) were small, which suggested that the marginal effect of exposure to one additional ACE was unlikely to produce a clinically significant change. However, in this sample the range of ACE exposure was 0–19, so the cumulative impact of heavy ACE exposure compared with light/no ACE exposure could still be clinically relevant. In the wider literature, reports on how ACE burden is linked to static QST parameters are also mixed. A recent systematic review and meta-analysis showed that traumatic life experiences (not limited to childhood) were associated with higher sensitivity in static QST; however, the authors did not present data for individual static QST parameters.^35^ Individual studies provide conflicting evidence; some have reported links between a higher ACE burden and greater sensitivity in thermal,^62^ mechanical parameters,^39^^,^^63^^,^^64^ or temporal summation/wind up parameters.^38^ However, the same studies and others report no links between a higher ACE burden and similar parameters.^39^^,^^63^^,^^65^^,^^66^ Many studies utilising QST have small sample sizes and so may lack the power to identify a small effect size.

Exploratory analyses involving dynamic QST parameters did not appear to show any relationship between ACE count and CPM effect in two modalities (PPT and HPT). This is broadly in line with the existing literature, although there are fewer studies testing this relationship. Three studies have reported no relationship between ACE burden and experimental CPM effect,^64^, ^65^, ^66^ whereas a fourth showed that ACE burden was only linked to higher pain sensitivity (weaker CPM effect) in participants who lived in more deprived neighbourhoods.^40^ Interpreting these findings is made more challenging by the difference in CPM protocols; for example, when considering the conditioning and test stimuli respectively, one study used a hand in a cold water bath with pressure applied to the lumbar paraspinal muscles,^40^ one study used a hand in a cold water bath with pressure applied to the contralateral thenar muscles,^66^ one used a pressure cuff on the left leg with a pressure cuff applied to the right leg,^64^ and one study used pressure on the non-dominant thumbnail with pressure applied to the dominant thumbnail.^65^ There were also variations in the timing and duration of stimuli, and in participant instructions. The comparability of these differing protocols remains uncertain; broader adoption of standardised methods would enhance consistency, reproducibility, and cross-study comparisons.

### Strengths and limitations

This study has several strengths. First, our study was designed and implemented with active PPI throughout. This was important given the sensitive nature of the subject material and the potential for questions or sensory tests to trigger unpleasant experiences. As a result, we are confident that participants felt safe throughout the study protocol (and this is reflected in the study acceptability questionnaire responses).

Second, we used a tool (the CAPE ACE questionnaire) that covered a broader range of ACEs (abuse, neglect, household challenges, and external challenges) than many commonly used tools (such as the Childhood Trauma Questionnaire). The impact of ACEs on long-term health outcomes is thought be attributable to prolonged exposure to stress,^67^ and so adopting a wider range of potentially stressful experiences is likely to give a better estimate of this cumulative burden.

Third, we followed validated QST protocols where they were available^52^^,^^53^^,^^55^ and used consensus recommendations for CPM protocols in the absence of a recognised standard.^56^

Fourth, all questionnaires and sensory tests were administered by the same investigator using the same equipment in the same environment, and so avoided inter-investigator differences in test administration and data recording.

This study has some limitations. First, ACEs were reported retrospectively by participants and so were potentially at risk of recall and reporting bias. This is a challenge faced in this field, as prospective measurements of ACEs are uncommon because of ethical issues. A recent cross-sectional study by Timmins and colleagues^68^ considered a negative control exposure (retrospective report of childhood sunburn) alongside retrospective reports of ACEs. Reporting of childhood sunburn had a negligible impact on severe chronic ‘all over’ body pain in adulthood, whereas reporting of ACEs had a large impact, which suggests that the observed association between ACEs and pain was not attributable to recall bias.^68^

Second, the ACE instrument used (the CAPE ACE questionnaire) is still being validated as part of the CAPE programme. However, it is based on validated tools (ACE-IQ,^49^ TDS,^50^ and TEC^51^), the face and content validity stages are completed, and the final validation exercise will provide a fully validated instrument to accompany this protocol in the future planned study.

Third, we used ACE count as a marker of cumulative stress experienced by an individual. This assumes that each ACE carries equal weight, whereas different ACEs may have different impacts, and these may vary between individuals. Some ACE questionnaires use scoring methods or estimates of frequency for individual ACEs in an attempt to address this, although our PPI group was clear in emphasising that these approaches do not accurately reflect the varied experiences of the individual.

Fourth, we were unable to recruit equally to the four study groups in our planned protocol and therefore did not perform our planned per-group analyses. Underrepresentation in the ‘chronic pain without multimorbidity’ category is likely a result of the close relationship between chronic pain and multimorbidity.^31^

Fifth, we did not distinguish between different chronic pain syndromes, considering chronic primary pain as a single diagnosis. This was a pragmatic decision to avoid further fragmenting an already small sample size, although there is also a large body of neuroimaging and brain circuitry research that suggests that different types of chronic pain have shared central pathways.^69^^,^^70^ However, it is also plausible that different chronic pain syndromes may have different sensory profiles,^71^ and a future definitive study may be able to consider this in more detail.

Sixth, although QST *z*-scores were calculated based on publicly available reference data,^55^ we were unable to determine whether our study sample was representative of the reference population. The *z*-score process accounts for age and gender, but it is possible that other factors (such as ethnicity) may influence components of the pain processing pathways.^72^, ^73^, ^74^

Seventh, as a feasibility study, this work was not powered to deliver definitive conclusions, and given the number of sensory tests included in both QST protocols, there is the potential for a type I error (a false positive). However, given the sensitivity of ACEs, it was essential to demonstrate that recruitment was feasible and study procedures did not cause distress before embarking on a larger-scale study. Analyses in a future study would incorporate corrections for multiple testing to reduce the likelihood of making a type I error. Our exploratory findings suggest that ACEs may be associated with chronic pain, multimorbidity, and altered sensory processing in some static QST parameters, but these should be considered preliminary data, pending confirmation from a larger study. Feasibility metrics and effect estimates from this study will guide protocol refinement, outcome prioritisation, sample size calculations, and recruitment timelines for a robust, appropriately powered, definitive study.

### Conclusions

This study demonstrated that assessing sensory processing in relation to ACEs among adults with chronic pain and multimorbidity was both feasible and acceptable. The exploratory findings suggest that ACE exposure may be associated with altered sensory processing in some mechanical static QST parameters, although the magnitudes of these relationships were modest. Dynamic QST parameters did not appear to be related to ACE exposure. An appropriately powered larger study would be valuable to interrogate these relationships further, although altered sensory processing is unlikely to be the only mechanism by which ACEs exert a long-term impact.

## Authors’ contributions

Study conception: DNSS, BHS, LAC

Study design and planning: all authors

Performed data collection and analysis, drafted the manuscript: DNSS

Interpretation of results: all authors

Reviewed and approved the final manuscript: all authors

## Funding

The authors are members of the Advanced Pain Discovery Platform (APDP) supported by UK Research & Innovation (UKRI), Versus Arthritis, and Eli Lilly. DNSS is a fellow on the Multimorbidity Doctoral Training Programme for Health Professionals, which is supported by the Wellcome Trust [223499/Z/21/Z]. BHS and LAC are supported by an APDP grant as part of the Partnership for Assessment and Investigation of Neuropathic Pain: Studies Tracking Outcomes, Risks and Mechanisms (PAINSTORM) consortium [MR/W002388/1]. TGH and LAC are supported by an APDP grant as part of the Consortium Against Pain in Equality [MR/W002566/1]. LM is supported by a UKRI Future Leaders Fellowship [MR/X035638/1]. The funding bodies had no role in study design, manuscript writing, or the decision to submit the manuscript for publication.

## Data availability statement

All data are available upon reasonable request from the corresponding author.

## Declarations of interest

All authors declare that they have no competing interests.

## References

[1] Madigan S., Deneault A., Racine N. (2023). Adverse childhood experiences: a meta-analysis of prevalence and moderators among half a million adults in 206 studies. World Psychiatry.

[2] Bellis M.A., Lowey H., Leckenby N., Hughes K., Harrison D. (2014). Adverse childhood experiences: retrospective study to determine their impact on adult health behaviours and health outcomes in a UK population. J Public Health Oxf Engl.

[3] Bussières A., Hancock M.J., Elklit A. (2023). Adverse childhood experience is associated with an increased risk of reporting chronic pain in adulthood: a systematic review and meta-analysis. Eur J Psychotraumatology.

[4] Felitti V.J., Anda R.F., Nordenberg D. (1998). Relationship of childhood abuse and household dysfunction to many of the leading causes of death in adults. The Adverse Childhood Experiences (ACE) Study. Am J Prev Med.

[5] Hardcastle K., Bellis M.A., Sharp C.A., Hughes K. (2020). Exploring the health and service utilisation of general practice patients with a history of adverse childhood experiences (ACEs): an observational study using electronic health records. BMJ Open.

[6] Hughes K., Bellis M.A., Hardcastle K.A. (2017). The effect of multiple adverse childhood experiences on health: a systematic review and meta-analysis. Lancet Public Health.

[7] Maniglio R. (2009). The impact of child sexual abuse on health: a systematic review of reviews. Clin Psychol Rev.

[8] Nicolson K.P., Mills S.E.E., Senaratne D.N.S., Colvin L.A., Smith B.H. (2023). What is the association between childhood adversity and subsequent chronic pain in adulthood? A systematic review. BJA Open.

[9] Rogers N.T., Power C., Pereira S.M.P. (2021). Child maltreatment, early life socioeconomic disadvantage and all-cause mortality in mid-adulthood: findings from a prospective British birth cohort. BMJ Open.

[10] Senaratne D.N.S., Koponen M., Barnett K.N. (2025). Impact of adverse childhood experiences on analgesia-related outcomes: a systematic review. Br J Anaesth.

[11] Senaratne D.N.S., Thakkar B., Smith B.H., Hales T.G., Marryat L., Colvin L.A. (2024). The impact of adverse childhood experiences on multimorbidity: a systematic review and meta-analysis. BMC Med.

[12] Yu J., Patel R.A., Haynie D.L. (2022). Adverse childhood experiences and premature mortality through mid-adulthood: a five-decade prospective study. Lancet Reg Health Am.

[13] Kim K., Yaffe K., Rehkopf D.H. (2023). Association of adverse childhood experiences with accelerated epigenetic aging in midlife. JAMA Netw Open.

[14] Jain P., Binder A., Chen B. (2023). The association of epigenetic age acceleration and multimorbidity at age 90 in the women’s health initiative. J Gerontol A Biol Sci Med Sci.

[15] Mau T., Blackwell T.L., Cawthon P.M. (2024). Muscle mitochondrial bioenergetic capacities are associated with multimorbidity burden in older adults: the Study of Muscle, Mobility and Aging (SOMMA). J Gerontol A Biol Sci Med Sci.

[16] Zang J.C.S., May C., Hellwig B. (2023). Proteome analysis of monocytes implicates altered mitochondrial biology in adults reporting adverse childhood experiences. Transl Psychiatry.

[17] Danese A., Baldwin J.R. (2017). Hidden wounds? Inflammatory links between childhood trauma and psychopathology. Annu Rev Psychol.

[18] Friedman E., Shorey C. (2019). Inflammation in multimorbidity and disability: an integrative review. Health Psychol Off J Div Health Psychol Am Psychol Assoc.

[19] Singleton S., Sneddon C., Bakina A., Lambert J.J., Hales T.G. (2023). Early-life adversity increases morphine tolerance and persistent inflammatory hypersensitivity through upregulation of δ opioid receptors in mice. Pain.

[20] Sheffler J.L., Meng Z., Sachs-Ericsson N., Caimary V.G., Patel J., Pickett S. (2025). Sleep quality as a critical pathway between adverse childhood experiences and multimorbidity and the impact of lifestyle. J Aging Health.

[21] Carlyle M., Broomby R., Simpson G. (2021). A randomised, double-blind study investigating the relationship between early childhood trauma and the rewarding effects of morphine. Addict Biol.

[22] Nakamoto K., Tokuyama S. (2023). Stress-induced changes in the endogenous opioid system cause dysfunction of pain and emotion regulation. Int J Mol Sci.

[23] Treede R.-D., Rief W., Barke A. (2019). Chronic pain as a symptom or a disease: the IASP Classification of Chronic Pain for the International Classification of Diseases (ICD-11). Pain.

[24] Rometsch C., Martin A., Junne F., Cosci F. (2025). Chronic pain in European adult populations: a systematic review of prevalence and associated clinical features. Pain.

[25] Sá K.N., Moreira L., Baptista A.F. (2019). Prevalence of chronic pain in developing countries: systematic review and meta-analysis. PAIN Rep.

[26] Zimmer Z., Fraser K., Grol-Prokopczyk H., Zajacova A. (2022). A global study of pain prevalence across 52 countries: examining the role of country-level contextual factors. Pain.

[27] Pitcher M.H., Von Korff M., Bushnell M.C., Porter L. (2019). Prevalence and profile of high-impact chronic pain in the United States. J Pain.

[28] Versus Arthritis (2017). Chronic pain in England: unseen, unequal, unfair. https://www.versusarthritis.org/about-arthritis/data-and-statistics/chronic-pain-in-england/.

[29] Nguyen H., Manolova G., Daskalopoulou C., Vitoratou S., Prince M., Prina A.M. (2019). Prevalence of multimorbidity in community settings: a systematic review and meta-analysis of observational studies. J Comorbidity.

[30] Hanlon P., McCallum M., Jani B.D., McQueenie R., Lee D., Mair F.S. (2020). Association between childhood maltreatment and the prevalence and complexity of multimorbidity: A cross-sectional analysis of 157,357 UK Biobank participants. J Comorbidity.

[31] McQueenie R., Jani B.D., Siebert S. (2021). Prevalence of chronic pain in LTCs and multimorbidity: a cross-sectional study using UK Biobank. J Multimorb Comorbidity.

[32] van Driel M.E.C., Huygen F.J.P.M., Rijsdijk M. (2024). Quantitative sensory testing: a practical guide and clinical applications. BJA Educ.

[33] Marcuzzi A., Wrigley P.J., Dean C.M., Adams R., Hush J.M. (2017). The long-term reliability of static and dynamic quantitative sensory testing in healthy individuals. Pain.

[34] Morris M.C., Goodin B.R., Bruehl S. (2023). Adversity type and timing predict temporal summation of pain in African-American adults. J Behav Med.

[35] Nanavaty N., Thompson C.G., Meagher M.W., McCord C., Mathur V.A. (2023). Traumatic life experience and pain sensitization: meta-analysis of laboratory findings. Clin J Pain.

[36] Rassu F.S., Luedke J.C., Nanavaty N., Mathur V.A., Meagher M.W. (2020). Greater mechanical temporal summation of pain in Latinx-Americans and the role of adverse life experiences. Pain Rep.

[37] Scarinci I.C., McDonald-Haile J., Bradley L.A., Richter J.E. (1994). Altered pain perception and psychosocial features among women with gastrointestinal disorders and history of abuse: a preliminary model. Am J Med.

[38] Sherman A.L., Morris M.C., Bruehl S., Westbrook T.D., Walker L.S. (2015). Heightened temporal summation of pain in patients with functional gastrointestinal disorders and history of trauma. Ann Behav Med Publ Soc Behav Med.

[39] Tesarz J., Eich W., Treede R.-D., Gerhardt A. (2016). Altered pressure pain thresholds and increased wind-up in adult patients with chronic back pain with a history of childhood maltreatment: a quantitative sensory testing study. Pain.

[40] Thomas P.A., Ditta P.V., Stocking S.Q. (2025). The effects of neighborhood disadvantage and adverse childhood experiences on conditioned pain modulation in adults with chronic low back pain. J Pain.

[41] Senaratne D.N.S., Smith B.H., Hales T.G., Marryat L., Colvin L.A. (2025). Impact of adverse childhood experiences on sensory thresholds in adults living with multimorbidity and chronic pain (the ACE-MAP study): protocol for an observational feasibility study. BMJ Open.

[42] (2024). The Consortium Against Pain inEquality (CAPE). https://dundee-cape.ac.uk.

[43] McKinstry B., Sullivan F.M., Vasishta S. (2017). Cohort profile: the Scottish Research register SHARE. A register of people interested in research participation linked to NHS data sets. BMJ Open.

[44] Ho I.S.S., Azcoaga-Lorenzo A., Akbari A. (2022). Measuring multimorbidity in research: Delphi consensus study. BMJ Med.

[45] Whitehead A.L., Julious S.A., Cooper C.L., Campbell M.J. (2016). Estimating the sample size for a pilot randomised trial to minimise the overall trial sample size for the external pilot and main trial for a continuous outcome variable. Stat Methods Med Res.

[46] Totton N., Lin J., Julious S., Chowdhury M., Brand A. (2023). A review of sample sizes for UK pilot and feasibility studies on the ISRCTN registry from 2013 to 2020. Pilot Feasibility Stud.

[47] Wynick D., Smith B.H., Bennett D., Macfarlane G.J. (2022). https://biobank.ndph.ox.ac.uk/showcase/ukb/docs/pain_questionnaire.pdf.

[48] Mosler F., Christogianni A., Singleton S. (2025). Assessing exposure to childhood adversity in adults: a systematic review of validated self-report childhood adversity questionnaires. Psychother Psychosom.

[49] Pace C.S., Muzi S., Rogier G., Meinero L.L., Marcenaro S. (2022). The Adverse Childhood Experiences–International Questionnaire (ACE-IQ) in community samples around the world: a systematic review (part I). Child Abuse Negl.

[50] Salokangas R.K.R., Schultze-Lutter F., Patterson P. (2016). Psychometric properties of the Trauma and Distress Scale, TADS, in an adult community sample in Finland. Eur J Psychotraumatol.

[51] Nijenhuis E.R.S., Van der Hart O., Kruger K. (2002). The psychometric characteristics of the Traumatic Experiences Checklist (TEC): first findings among psychiatric outpatients. Clin Psychol Psychother.

[52] Rolke R., Baron R., Maier C. (2006). Quantitative sensory testing in the German Research Network on Neuropathic Pain (DFNS): standardized protocol and reference values. Pain.

[53] Rolke R., Magerl W., Campbell K.A. (2006). Quantitative sensory testing: a comprehensive protocol for clinical trials. Eur J Pain.

[54] Sekhon M., Cartwright M., Francis J.J. (2022). Development of a theory-informed questionnaire to assess the acceptability of healthcare interventions. BMC Health Serv Res.

[55] Magerl W., Krumova E.K., Baron R., Tölle T., Treede R.-D., Maier C. (2010). Reference data for quantitative sensory testing (QST): refined stratification for age and a novel method for statistical comparison of group data. Pain.

[56] Yarnitsky D., Bouhassira D., Drewes A.M. (2015). Recommendations on practice of conditioned pain modulation (CPM) testing. Eur J Pain.

[57] Byeon S., Lee W. (2023). Directed acyclic graphs for clinical research: a tutorial. J Minim Invasive Surg.

[58] Tennant P.W.G., Murray E.J., Arnold K.F. (2020). Use of directed acyclic graphs (DAGs) to identify confounders in applied health research: review and recommendations. Int J Epidemiol.

[59] Galobardes B., Shaw M., Lawlor D.A., Lynch J.W., Smith G.D. (2006). Indicators of socioeconomic position (part 1). J Epidemiol Community Health.

[60] Firth D. (1993). Bias reduction of maximum likelihood estimates. Biometrika.

[61] National Institute of Health and Care Excellence (2025). British National Formulary. https://bnf.nice.org.uk/.

[62] Zarchev M., Kamperman A.M., de Leeuw T.G. (2025). The association between childhood maltreatment and pain sensitivity in a high-risk adolescent population. J Pain.

[63] Levy Gigi E., Rachmani M., Defrin R. (2024). The relationship between traumatic exposure and pain perception in children: the moderating role of posttraumatic symptoms. Pain.

[64] Lucas R., Talih M., Soares S., Fraga S. (2024). Bullying involvement and physical pain between ages 10 and 13 years: reported history and quantitative sensory testing in a population-based cohort. J Pain.

[65] Pierce J., Hassett A.L., Brummett C.M. (2021). Characterizing pain and generalized sensory sensitivity according to trauma history among patients with knee osteoarthritis. Ann Behav Med.

[66] Zehetmeier K.F., Fröhlich M.K., Schilder A. (2023). The association between adverse childhood experiences and peripartal pain experience. Pain.

[67] Nelson C.A., Bhutta Z.A., Harris N.B., Danese A., Samara M. (2020). Adversity in childhood is linked to mental and physical health throughout life. BMJ.

[68] Timmins K.A., Hales T.G., Macfarlane G.J., Consortium Against Pain InEquality (CAPE) investigators and Chronic Pain Advisory Group (2025). Childhood maltreatment and chronic ‘all over’ body pain in adulthood: a counterfactual analysis using UK Biobank. Pain.

[69] Barroso J., Branco P., Apkarian A.V. (2021). Brain mechanisms of chronic pain: critical role of translational approach. Transl Res J Lab Clin Med.

[70] Tan L.L., Kuner R. (2021). Neocortical circuits in pain and pain relief. Nat Rev Neurosci.

[71] Vollert J., Maier C., Attal N. (2017). Stratifying patients with peripheral neuropathic pain based on sensory profiles: algorithm and sample size recommendations. Pain.

[72] Ahn H., Weaver M., Lyon D.E. (2017). Differences in clinical pain and experimental pain sensitivity between Asian Americans and Whites with knee osteoarthritis. Clin J Pain.

[73] Li R., Holley A.L., Palermo T.M., Ohls O., Edwards R.R., Rabbitts J.A. (2023). Feasibility and reliability of a quantitative sensory testing protocol in youth with acute musculoskeletal pain post-surgery or post-injury. Pain.

[74] Ostrom C., Bair E., Maixner W. (2017). Demographic predictors of pain sensitivity: results from the OPPERA study. J Pain.

