# Supplementary material for: The impact of adverse childhood experiences on sensory thresholds in adults living with multimorbidity and chronic pain: an observational feasibility study
Source: BJA Open. 2026 Mar 10;17:100545. doi: 10.1016/j.bjao.2026.100545 (PMC12995835; doi:10.1016/j.bjao.2026.100545)
Supplement: Supplementary file 1 [file mmc1.docx]

The impact of adverse childhood experiences on sensory thresholds in adults living with multimorbidity and chronic pain (the ACE-MAP study): an observational feasibility study – supplementary material

Dhaneesha N.S. Senaratne, Blair H. Smith, Tim G. Hales, Louise Marryat, Lesley A. Colvin

[Figure S1: Directed-acyclic graph (DAG) outlining theoretical framework 2](#_Toc206689224)

[Figure S2: Self-reported ACE prevalence 3](#_Toc206689225)

[Table S1: Chronic pain status on static QST parameters 4](#_Toc206689226)

[Table S2: Multimorbidity status on static QST parameters 5](#_Toc206689227)

[Table S3: Chronic pain status on dynamic QST 6](#_Toc206689228)

[Table S4: Multimorbidity status on dynamic QST 7](#_Toc206689229)

# Figure S1: Directed-acyclic graph (DAG) outlining theoretical framework

Figure S1: Directed acyclic graph (DAG) demonstrating the assumptions used for analysis of the impact of ACEs on quantitative sensory testing (QST) outcomes. The resulting sufficient adjustment set (coloured blue) is age, sex, ethnicity, and childhood SES. In these analyses, education was used as a surrogate for childhood SES. ACE: adverse childhood experience, MM: multimorbidity, SES: socioeconomic status.

# Figure S2: Self-reported ACE prevalence

Figure S2: The prevalence of adverse childhood experiences in this sample, as reported by participants.

# Table S1: Chronic pain status on static QST parameters

| **QST parameter** | **Raw values** | | | **Z-scores** | | |
| --- | --- | --- | --- | --- | --- | --- |
|  | **β** | **SE** | **95% CI** | **β** | **SE** | **95% CI** |
| **CDT** (Δ°C from 32°C) | 0.03 | 0.24 | -0.43 to 0.49 | -0.06 | 0.18 | -0.41 to 0.28 |
| **WDT** (Δ°C from 32°C) | -0.30 | 0.32 | -0.93 to 0.33 | -0.45 | 0.26 | -0.96 to 0.06 |
| **TSL** (°C) | -0.56 | 0.43 | -1.41 to 0.29 | -0.27 | 0.15 | -0.56 to 0.03 |
| **CPT** (°C) | 2.48 | 2.39 | -2.20 to 7.16 | 0.34 | 0.29 | -0.23 to 0.91 |
| **HPT** (°C) | -1.05 | 0.99 | -2.99 to 0.89 | 0.39^#^ | 0.37 | -0.34 to 1.11 |
| **MDT** (mN) | -0.21 | 4.35 | -8.74 to 8.31 | 0.26 | 0.33 | -0.39 to 0.91 |
| **MPT** (mN) | 9.66 | 27.22 | -43.69 to 63.00 | -0.26^#^ | 0.39 | -1.02 to 0.50 |
| **MPS** (rating 0-100) | 0.87 | 1.04 | -1.17 to 2.91 | 0.36 | 0.43 | -0.48 to 1.20 |
| **DMA** (rating 0-100) | -0.01 | 0.03 | -0.08 to 0.06 | 0.10 | 2.16 | -4.13 to 4.34 |
| **WUR** (ratio) | -0.54 | 1.15 | -2.80 to 1.72 | -0.45 | 0.40 | -1.24 to 0.34 |
| **VDT** (score 0-8) | -0.02 | 0.05 | -0.11 to 0.06 | -0.06 | 0.11 | -0.27 to 0.15 |
| **PPT** (kPa) | -5.42 | 31.54 | -67.24 to 56.41 | 0.18^#^ | 0.52 | -0.83 to 1.19 |

Table S1: The impact of chronic pain status on static QST parameters, reported as raw values and z-scores. Each line reports two separate linear regression models (for raw values and z-scores respectively) adjusted for age, sex at birth, ethnicity, education, and ace count (covariate model parameters not shown). ^#^Z-scores for HPT, MPT, and PPT were multiplied by -1 so that all positive z-scores reflect gain of function (higher sensitivity to stimuli) and all negative scores reflect loss of function (lower sensitivity to stimuli). ACE: adverse childhood experience, CDT: cold detection threshold, CI: confidence interval, CPT: cold pain threshold, DMA: dynamic mechanical allodynia, HPT: heat pain threshold, MDT: mechanical detection threshold, MPS: mechanical pain sensitivity, MPT: mechanical pain threshold, PHS: paradoxical heat stimuli, PPT: pressure pain threshold, SE: standard error, TSL: thermal sensory limens, WDT: warm detection threshold, WUR: wind up ratio, VDT: vibration detection threshold.

# Table S2: Multimorbidity status on static QST parameters

| **QST parameter** | **Raw values** | | | **Z-scores** | | |
| --- | --- | --- | --- | --- | --- | --- |
|  | **β** | **SE** | **95% CI** | **β** | **SE** | **95% CI** |
| **CDT** (Δ°C from 32°C) | 0.12 | 0.19 | -0.25 to 0.49 | -0.19 | 0.14 | -0.46 to 0.08 |
| **WDT** (Δ°C from 32°C) | -0.02 | 0.26 | -0.53 to 0.49 | -0.12 | 0.21 | -0.54 to 0.30 |
| **TSL** (°C) | 0.12 | 0.37 | -0.60 to 0.84 | -0.04 | 0.13 | -0.29 to 0.20 |
| **CPT** (°C) | 2.45 | 1.88 | -1.25 to 6.14 | 0.32 | 0.23 | -0.14 to 0.77 |
| **HPT** (°C) | -0.97 | 0.79 | -2.51 to 0.57 | 0.37^#^ | 0.30 | -0.21 to 0.95 |
| **MDT** (mN) | 3.22 | 3.77 | -4.16 to 10.60 | 0.34 | 0.29 | -0.23 to 0.91 |
| **MPT** (mN) | -40.19 | 21.60 | -82.51 to 2.14 | 0.59^#^ | 0.32 | -0.03 to 1.21 |
| **MPS** (rating 0-100) | 1.78 | 0.92 | -0.02 to 3.58 | 0.64 | 0.36 | -0.06 to 1.34 |
| **DMA** (rating 0-100) | 0.05 | 0.03 | -0.01 to 0.10 | 2.85 | 1.81 | -0.70 to 6.40 |
| **WUR** (ratio) | 0.37 | 0.93 | -1.44 to 2.19 | 0.08 | 0.33 | -0.56 to 0.72 |
| **VDT** (score 0-8) | 0.02 | 0.04 | -0.05 to 0.09 | 0.05 | 0.09 | -0.12 to 0.21 |
| **PPT** (kPa) | -27.53 | 25.16 | -76.84 to 21.78 | 0.58^#^ | 0.41 | -0.24 to 1.39 |

Table S2: The impact of multimorbidity status on static QST parameters, reported as raw values and z-scores. Each line reports two separate linear regression models (for raw values and z-scores respectively) adjusted for age, sex at birth, ethnicity, education, and ace count (covariate model parameters not shown). ^#^Z-scores for HPT, MPT, and PPT were multiplied by -1 so that all positive z-scores reflect gain of function (higher sensitivity to stimuli) and all negative scores reflect loss of function (lower sensitivity to stimuli). ACE: adverse childhood experience, CDT: cold detection threshold, CI: confidence interval, CPT: cold pain threshold, DMA: dynamic mechanical allodynia, HPT: heat pain threshold, MDT: mechanical detection threshold, MPS: mechanical pain sensitivity, MPT: mechanical pain threshold, PHS: paradoxical heat stimuli, PPT: pressure pain threshold, SE: standard error, TSL: thermal sensory limens, WDT: warm detection threshold, WUR: wind up ratio, VDT: vibration detection threshold.

# Table S3: Chronic pain status on dynamic QST

| **QST parameter** | **Absolute CPM effect** | | | **Percentage CPM effect** | | |
| --- | --- | --- | --- | --- | --- | --- |
|  | **β** | **SE** | **95% CI** | **β** | **SE** | **95% CI** |
| **HPT** | -0.30 | 1.12 | -2.50 to 1.90 | -1.13 | 2.86 | -6.75 to 4.48 |
| **PPT** | 28.08 | 20.39 | -11.90 to 68.05 | 8.74 | 9.00 | -8.90 to 26.38 |

Table S3: The impact of chronic pain status on dynamic QST, reported as absolute CPM effect and percentage CPM effect. Each line reports two separate linear regression models (for absolute and percentage CPM effects respectively) adjusted for age, sex at birth, ethnicity, education, and number of ACEs reported (covariate model parameters not shown). ACE: adverse childhood experience, β: beta regression coefficient, CI: confidence interval, CPM: conditioned pain modulation, QST: quantitative sensory testing, SE: standard error.

# Table S4: Multimorbidity status on dynamic QST

| **QST parameter** | **Absolute CPM effect** | | | **Percentage CPM effect** | | |
| --- | --- | --- | --- | --- | --- | --- |
|  | **β** | **SE** | **95% CI** | **β** | **SE** | **95% CI** |
| **HPT** | 1.32 | 1.06 | -0.76 to 3.39 | 3.73 | 2.69 | -1.54 to 9.00 |
| **PPT** | 2.61 | 19.53 | -35.67 to 40.89 | 3.61 | 8.61 | -13.25 to 20.48 |

Table S4: The impact of multimorbidity status on dynamic QST, reported as absolute CPM effect and percentage CPM effect. Each line reports two separate linear regression models (for absolute and percentage CPM effects respectively) adjusted for age, sex at birth, ethnicity, education, and number of ACEs reported (covariate model parameters not shown). ACE: adverse childhood experience, β: beta regression coefficient, CI: confidence interval, CPM: conditioned pain modulation, QST: quantitative sensory testing, SE: standard error.
